# Supplementary material for: Biomarker potential of repetitive-element transcriptome in lung cancer
Source: PeerJ. 2019 Dec 19;7:e8277. doi: 10.7717/peerj.8277 (PMC6925957; doi:10.7717/peerj.8277)
Supplement: Figure S1 — For LUAD (upper panel) and SCLC (lower panel), the logFCpP (ordinate) of all REs in each patient (abscissa) are represented. logFC = 0 is marked with a green line to show that the overall level of RE expression can be considered unchanged in each patient after an oncogenic process. [file peerj-07-8277-s001.pdf]

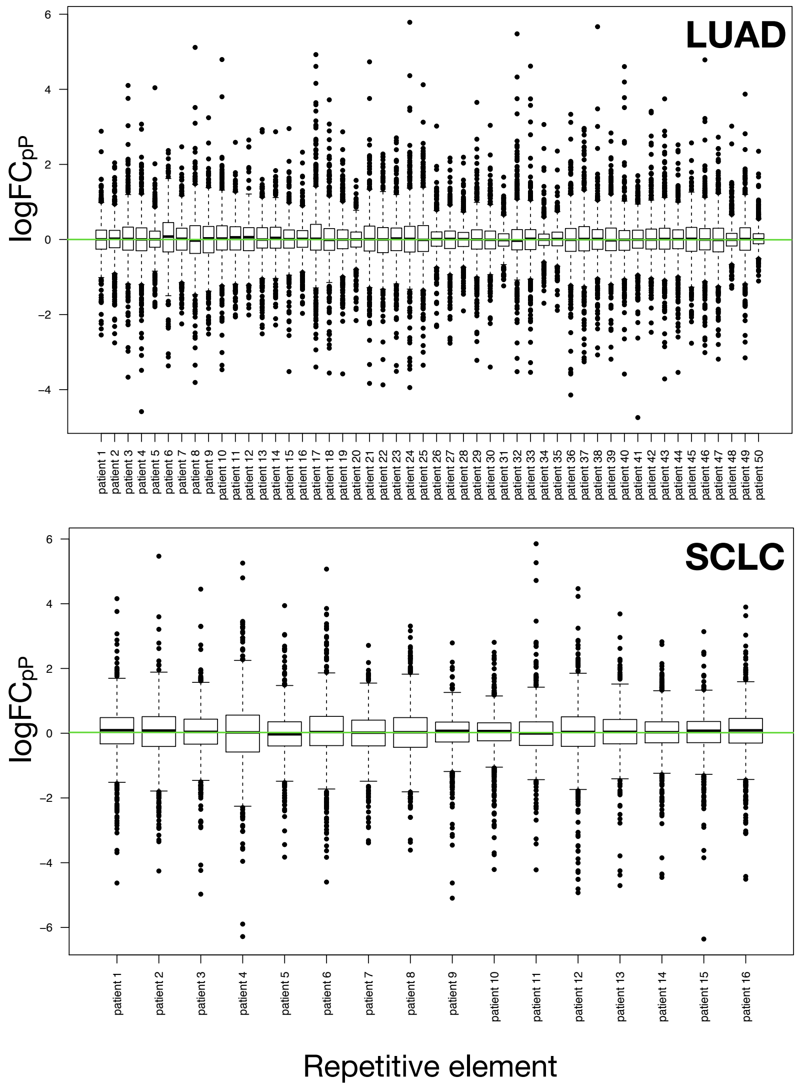

Figure S1. Overall RE expression change per patient. For LUAD (upper panel) and SCLC (lower panel), the $logFC_{pP}$ (ordinate) of all REs in each patient (abscissa) are represented. $logFC = 0$ is marked with a green line to show that the overall level of RE expression can be considered unchanged in each patient after an oncogenic process.
