# Supplementary material for: Biomarker potential of repetitive-element transcriptome in lung cancer
Source: PeerJ. 2019 Dec 19;7:e8277. doi: 10.7717/peerj.8277 (PMC6925957; doi:10.7717/peerj.8277)
Supplement: Table S1 — Positive logFCOE values indicate up-regulation in normal cells of SCLC, while negative values indicate up-regulation in normal cells of LUAD samples. [file peerj-07-8277-s002.pdf]

Table S1. Name, class and family of differentially expressed REs when normal lung from SCLC is compared with normal lung from LUAD. Positive $logFC_{OE}$ values indicate up-regulation in normal cells of SCLC, while negative values indicate up-regulation in normal cells of LUAD samples.

RE	Class	Family	$logFC_{OE}$	FDR
7SLRNA	srpRNA	srpRNA	2.33	3.56e-33
LTR7A	LTR	ERV1	2.25	5.22e-29
7SK	RNA	RNA	2.16	1.98e-26
MER11B	LTR	ERVK	1.97	4.13e-24
ERV24B_Prim-int	LTR	ERV1	1.73	1.97e-11
LTR6A	LTR	ERV1	1.52	1.98e-33
UCON50	Unknown	Unknown	1.34	1.90e-09
LTR25-int	LTR	ERV1	1.33	1.84e-51
LTR54B	LTR	ERV1	1.28	2.77e-19
MER65-int	LTR	ERV1	1.25	1.76e-10
L1PA6	LINE	L1	1.17	3.81e-13
AluYb8	SINE	Alu	1.17	4.03e-20
MER134	DNA?	DNA?	1.15	2.10e-17
LTR82A	LTR	ERVL	1.10	1.98e-26
UCON6	Unknown	Unknown	1.09	3.61e-24
L1P4d	LINE	L1	1.07	6.47e-07
MER75A	DNA	PiggyBac	1.06	5.63e-07
Eulor2C	DNA?	DNA?	1.06	0.00
MER132	DNA	TcMar-Pogo	1.05	0.00
Ricksha_a	DNA	MULE-MuDR	1.04	7.43e-08
L1M4c	LINE	L1	1.04	2.43e-33
UCON39	DNA	TcMar-Tigger	1.03	7.54e-17
LTR47A2	LTR	ERVL	1.00	1.68e-12
LTR06	LTR	ERV1	1.00	3.02e-08
MamGypsy2-LTR	LTR	Gypsy	1.00	8.44e-22
UCON35	Unknown	Unknown	-1.39	3.39e-12
HERVK11-int	LTR	ERVK	-1.16	2.52e-19
UCON23	DNA	hAT-Tip100?	-1.08	1.50e-13
UCON51	LTR?	LTR?	-1.07	2.48e-07
LTR23-int	LTR	ERV1	-1.06	3.82e-10
