# Supplementary material for: Biomarker potential of repetitive-element transcriptome in lung cancer
Source: PeerJ. 2019 Dec 19;7:e8277. doi: 10.7717/peerj.8277 (PMC6925957; doi:10.7717/peerj.8277)
Supplement: Table S2 — These data were used in Fig. 3. [file peerj-07-8277-s003.pdf]

Table S2. Name, class and family of differentially expressed REs in LUAD together with their $logFC_{OE}$ and their statistical significance as an FDR. These data were used in Fig. 3.

RE	Class	Family	$logFC_{OE}$	FDR
HERVL18-int	LTR	ERVL	2.0	1.9e-29
LTR4	LTR	ERV1	1.4	1.5e-34
LTR18A	LTR	ERVL	1.4	2.2e-27
ALR_Alpha	Satellite	centr	1.3	1.9e-19
HERVK11D-int ●	LTR	ERVK	1.1	5.6e-12
MER65-int	LTR	ERV1	1.1	9.0e-09
HERV3-int	LTR	ERV1	1.1	3.5e-27
UCON80	DNA	hAT?	-1.1	3.0e-13
LTR77	LTR	ERV1	-1.1	4.5e-26
L1MEa	LINE	L1	-1.2	1.2e-35
UCON34	Unknown	Unknown	-1.3	6.5e-24
MER126	DNA	DNA	-1.3	2.3e-32
UCON26	Unknown	Unknown	-1.4	6.1e-35
AluYg6	SINE	Alu	-1.5	1.0e-52
LTR18B	LTR	ERVL	-2.1	8.8e-59

●: Potential LUAD-specific biomarker
