# Supplementary material for: Biomarker potential of repetitive-element transcriptome in lung cancer
Source: PeerJ. 2019 Dec 19;7:e8277. doi: 10.7717/peerj.8277 (PMC6925957; doi:10.7717/peerj.8277)
Supplement: Table S3 [file peerj-07-8277-s004.pdf]

Table S3. Expression change by RE family and class in LUAD. The single statistically significant family is marked with an asterisk (*).

**Family**	LogFC	FDR
centr *	1.02108481226983	1.51204509066979e-16
Unknown	-0.640893817333789	2.37698556392978e-34
telo	0.587989944258578	2.84100129717064e-13
DNA	-0.561270255150791	5.89860140844115e-24
hAT-Tag1	-0.548271509514666	1.70909494388762e-11
Dong-R4	-0.461306954287302	4.17593117416971e-08
hAT?	-0.437732777700732	3.45017075072195e-12
hAT	-0.42999101593034	2.33676188933125e-06
MIR	-0.423120296032459	3.09053134460215e-25
Merlin	0.423086446506984	4.71890722915744e-09
5S-Deu-L2	-0.413242910565126	1.70909494388762e-11
PiggyBac?	-0.404708115737808	4.68348532916706e-12
DNA?	-0.378000405050932	1.3955267519979e-18
tRNA-Deu	-0.302739936926857	1.7229732452279e-06
ERVK	0.283015564829794	3.22101423139124e-11
ERV1?	0.273221171008698	1.28571908180268e-07
hAT-Tip100	-0.258223271756418	2.91978768556969e-17
ERV1	0.249986608138221	2.7030034760509e-17
Helitron	0.247775049966194	1.0938501827259e-07
Satellite	0.244078933594483	0.000239247375944141
TcMar-Mariner	-0.239911392034992	1.51204509066979e-16
RTE-BovB	-0.213923614774419	1.6375418852645e-09
hAT-Ac	-0.210967568481547	9.44417060099763e-06
tRNA-RTE	-0.181183174801822	8.16227906510096e-08
MULE-MuDR	-0.157069061695668	0.00802263738351642
TcMar	-0.153612421169179	0.0451093046449653
L1	0.138252666345294	5.72156164376267e-10
ERVL-MaLR	0.12255463413171	5.08892198918431e-09
PiggyBac	0.111924829071776	0.0162061967915319
LTR	-0.109385877206312	0.00774513357306022
hAT-Blackjack	-0.102667254007691	0.00220490202258751
L2	0.0994118716441634	1.28571908180268e-07
Gypsy	-0.0804251117181345	0.0162061967915319
ERVL	0.0708430910125176	0.00108853834207793
LTR?	0.0652496638148965	0.0281134479234682
SVA	0.0616963154136462	0.196997547743618
CR1	-0.0533832202176372	0.00887211655772734
RNA	0.0518603237429111	0.573219739251327
TcMar-Tigger	0.0427522561589533	0.0102933365574504
Penelope	0.04145445698786	0.361781516406121
scRNA	0.0401766895232367	0.275577045636525
hAT-Tip100?	-0.0388916266050875	0.366118286547699
Gypsy?	0.036401481841554	0.471533011982807
srpRNA	0.0329421066037467	0.65995585961405
Alu	-0.0265189273584598	0.275577045636525
Helitron?	0.0264441075362418	0.718392136946153
RTE-X	0.0140616824297084	0.626977386032949
ERVL?	-0.00798590354778424	0.843698739800074
hAT-Charlie	0.00737844872113679	0.626977386032949
TcMar-Tc2	0.00548425450210184	0.848291930984676
**Class**	LogFC	FDR
Unknown	-0.682423513998543	3.1957018464701e-33
Satellite	0.504615728386989	9.27171444710761e-12
DNA?	-0.418671709965699	3.87007447142907e-18
SINE	-0.219206625716784	3.94612281090641e-23
RC	0.211102469741813	1.49573598665127e-05
LTR	0.125537324640516	1.12801119510303e-12
LINE	0.07148451583775	1.81750633992914e-06
DNA	-0.0610607616989778	0.000984893601365505
LTR?	0.0252604028615444	0.570392798693321
Retroposon	0.0212843756478532	0.82085838889315
RNA	0.015061020268293	0.976514125315192
RC?	-0.0107598645453814	0.976514125315192
srpRNA	-0.00770008133231628	0.976514125315192
scRNA	0.0010480187031418	0.977382102145543
