# Supplementary material for: Biomarker potential of repetitive-element transcriptome in lung cancer
Source: PeerJ. 2019 Dec 19;7:e8277. doi: 10.7717/peerj.8277 (PMC6925957; doi:10.7717/peerj.8277)
Supplement: Table S4 — These data were used in Figs. 4 and 5. [file peerj-07-8277-s005.pdf]

Table S4. Name, class and family of differentially down-regulated REs in SCLC together with their $logFC_{OE}$ and their statistical significance as an FDR. These data were used in Figures 4 and 5.

RE	Class	Family	$logFC_{OE}$	FDR
LTR7B •	LTR	ERV1	-3.8	1.9e-130
LTR18B •	LTR	ERVL	-2.8	1.8e-22
LTR9C •	LTR	ERV1	-2.7	8.3e-41
UCON26 •	Unknown	Unknown	-2.5	9.0e-44
LTR54B •	LTR	ERV1	-2.5	1.9e-41
LTR1B •	LTR	ERV1	-2.3	1.9e-96
HERV1_LTRc •	LTR	ERV1	-2.2	4.1e-25
UCON80 •	DNA	hAT?	-2.1	9.1e-22
MER65-int	LTR	ERV1	-2.0	1.2e-07
Ricksha_a •	DNA	MULE-MuDR	-1.9	3.2e-23
ERVL47-int •	LTR	ERVL	-1.7	5.6e-24
ERV24B_Prim-int	LTR	ERV1	-1.7	1.1e-06
MIRc •	SINE	MIR	-1.7	9.0e-39
UCON34	Unknown	Unknown	-1.7	1.1e-16
LTR2C •	LTR	ERV1	-1.6	1.4e-26
MER91A •	DNA	hAT-Tip100	-1.6	2.4e-14
UCON14	DNA?	DNA?	-1.5	7.5e-09
HERVI-int	LTR	ERV1	-1.5	1.2e-15
MLT1E •	LTR	ERVL-MaLR	-1.5	1.6e-27
MER53	DNA	hAT	-1.5	1.2e-07
MER126	DNA	DNA	-1.5	2.6e-14
UCON67	Unknown	Unknown	-1.4	5.5e-14
AluYg6 •	SINE	Alu	-1.4	7.8e-15
MIR •	SINE	MIR	-1.4	6.0e-38
AluYk2	SINE	Alu	-1.4	2.1e-19
HERVE-int •	LTR	ERV1	-1.4	6.7e-24
MIR3 •	SINE	MIR	-1.4	2.9e-25
LTR39	LTR	ERV1	-1.4	1.8e-06
L1MEa	LINE	L1	-1.4	9.4e-09
MER61F	LTR	ERV1	-1.3	1.3e-13
MER63A	DNA	hAT-Blackjack	-1.3	2.2e-16
LTR22A	LTR	ERVK	-1.3	4.9e-13
LTR27B •	LTR	ERV1	-1.2	3.4e-19
MER39	LTR	ERV1	-1.2	2.4e-13
MamRep564 •	Unknown	Unknown	-1.2	4.8e-09
Charlie26a	DNA	hAT-Charlie	-1.2	3.5e-23
MER57C1	LTR	ERV1	-1.1	2.8e-12
UCON39	DNA	TcMar-Tigger	-1.1	4.4e-07
L1M2a1	LINE	L1	-1.1	5.1e-12
HERV1_LTRd	LTR	ERV1	-1.1	5.9e-09
MIRb •	SINE	MIR	-1.1	3.8e-30
LTR57 •	LTR	ERVL	-1.1	9.1e-11
CR1_Mam •	LINE	CR1	-1.1	8.0e-19
LTR81A •	LTR	Gypsy	-1.1	1.8e-19
MER57D •	LTR	ERV1	-1.1	4.6e-18
LTR77	LTR	ERV1	-1.1	3.3e-07
Zaphod3 •	DNA	hAT-Tip100	-1.0	1.2e-12
ORSL-2a	DNA	hAT-Tip100	-1.0	2.1e-06
MER57F	LTR	ERV1	-1.0	7.1e-09
LTR10E	LTR	ERV1	-1.0	3.9e-08

•: Potential SCLC-specific biomarker
