# Supplementary material for: Biomarker potential of repetitive-element transcriptome in lung cancer
Source: PeerJ. 2019 Dec 19;7:e8277. doi: 10.7717/peerj.8277 (PMC6925957; doi:10.7717/peerj.8277)
Supplement: Table S5 — These data were used in Figs. 4 and 5. [file peerj-07-8277-s006.pdf]

Table S5. Name, class and family of differentially up-regulated REs in SCLC together with their $logFC_{OE}$ and their statistical significance as an FDR. These data were used in Figures 4 and 5.

differentially expressed RE	Class	Family	$logFC_{OE}$	FDR
MLT2B5	LTR	ERVL	2.6	1.1e-13
HERVL18-int	LTR	ERVL	2.1	5.0e-10
LTR18A	LTR	ERVL	1.8	6.3e-12
REP522	Satellite	telo	1.8	1.7e-09
Charlie11 •	DNA	hAT-Charlie	1.7	1.3e-27
LTR12F	LTR	ERV1	1.7	2.2e-13
SST1	Satellite	centr	1.6	1.3e-13
HERVS71-int	LTR	ERV1	1.5	2.6e-14
UCON88•	Unknown	Unknown	1.3	6.6e-06
BSR_Beta	Satellite	Satellite	1.3	6.8e-16
MER51E •	LTR	ERV1	1.2	4.1e-17
L1M3a •	LINE	L1	1.2	4.8e-13
Tigger5 •	DNA	TcMar-Tigger	1.1	3.3e-15
MER75A	DNA	PiggyBac	1.1	1.3e-07
UCON8	DNA	DNA	1.1	9.0e-09
HERV1_I-int	LTR	ERV1	1.1	8.7e-05
LTR25 •	LTR	ERV1	1.1	1.4e-14
LTR21A	LTR	ERV1	1.0	2.3e-05
LTR12D	LTR	ERV1	1.0	8.4e-07
LTR49-int •	LTR	ERV1	1.0	5.2e-16
L1PA12 •	LINE	L1	1.0	8.1e-12

•: Potential SCLC-specific biomarker
