# Supplementary material for: Biomarker potential of repetitive-element transcriptome in lung cancer
Source: PeerJ. 2019 Dec 19;7:e8277. doi: 10.7717/peerj.8277 (PMC6925957; doi:10.7717/peerj.8277)
Supplement: Table S6 [file peerj-07-8277-s007.pdf]

**Table S6.** Expression change by RE family and class in SCLC. The statistically significant families and class are marked with an asterisk (*).

| Family | LogFC | FDR |
|---|---|---|
| telo * | 1.34854671086538 | 0.0000365481574358304 |
| MIR * | -1.3261688614074 | 1.3884123315054e-50 |
| centr * | 1.05324492837102 | 2.42267167955532e-06 |
| hAT * | -1.02930627386871 | 2.42267167955532e-06 |
| Satellite | 0.909745955106403 | 1.46940002050509e-08 |
| hAT? | -0.766581450026728 | 2.42267167955532e-06 |
| Unknown | -0.552571465313136 | 0.00014476417515476 |
| hAT-Tip100 | -0.509791996488389 | 1.42144278834333e-07 |
| Helitron | 0.413701520765789 | 0.00893010771018864 |
| TcMar-Tc2 | 0.367602720548 | 0.00331733794267837 |
| hAT-Tag1 | 0.363556845440107 | 0.0963676176539064 |
| PiggyBac | 0.346499808908819 | 0.0109178279770229 |
| Merlin | 0.341344257307743 | 0.156770545549572 |
| hAT-Blackjack | -0.329763239017602 | 0.00600238078728352 |
| tRNA-Deu | -0.285747490223139 | 0.101783103582224 |
| srpRNA | 0.258615242701252 | 0.532206509664075 |
| LTR | -0.251722580028142 | 0.156770545549572 |
| ERVK | 0.248250367060801 | 0.0446314223741959 |
| ERV1? | -0.247045319676052 | 0.141765597589778 |
| CR1 | -0.23909345344551 | 0.00425465364991257 |
| RTE-BovB | -0.225802900375445 | 0.0830474849974313 |
| ERV1 | -0.211996988068223 | 0.0143669730312461 |
| DNA | -0.200541253704206 | 0.205403014944661 |
| Penelope | 0.197204842443767 | 0.302764586297193 |
| DNA? | 0.188073343054497 | 0.245618119339655 |
| Gypsy | 0.186088596198932 | 0.254386374190543 |
| Helitron? | 0.183148874162789 | 0.320915182784707 |
| LTR? | 0.156350798058122 | 0.24736784329282 |
| Alu | -0.150679864141522 | 0.0809298073965674 |
| MULE-MuDR | -0.149056110264191 | 0.320915182784707 |
| TcMar-Tigger | 0.143183695583773 | 0.0681266819071531 |
| L2 | -0.139095071094924 | 0.101783103582224 |
| ERVL? | -0.137261710285251 | 0.31669801926347 |
| Gypsy? | 0.132942922272655 | 0.532206509664075 |
| tRNA-RTE | -0.131693324227164 | 0.337813649829251 |
| RTE-X | -0.106885571631967 | 0.411291624353563 |
| RNA | -0.101641194551597 | 0.761030057928728 |
| TcMar | -0.0966746515967049 | 0.718277544705426 |
| scRNA | -0.088792512065464 | 0.590931997035589 |
| ERVL-MaLR | 0.0881273412465159 | 0.250074331780613 |
| L1 | 0.0860287993663588 | 0.254386374190543 |
| 5S-Deu-L2 | -0.0853981789182889 | 0.639756878021122 |
| SVA | -0.0846774146834285 | 0.639956367603783 |
| hAT-Tip100? | -0.0740951295365135 | 0.639956367603783 |
| hAT-Ac | -0.0614123616718557 | 0.643251914778013 |
| Dong-R4 | 0.0607717447179489 | 0.735820162917559 |
| ERVL | 0.0400310660989352 | 0.643251914778013 |
| TcMar-Mariner | 0.0392456665434142 | 0.735820162917559 |
| hAT-Charlie | -0.0251788579976489 | 0.718277544705426 |
| PiggyBac? | 0.0147638290216924 | 0.919150949917058 |
| **Class** | LogFC | FDR |
| Satellite * | 1.13241956464061 | 2.57583142433929e-11 |
| SINE | -0.509932859935055 | 1.94196950632935e-21 |
| RC | 0.491867461627475 | 0.00782212265726685 |
| Unknown | -0.472629622743312 | 0.00782212265726685 |
| srpRNA | 0.332201800283961 | 0.360910107515738 |
| DNA? | 0.268027204514469 | 0.176155270922065 |
| RC? | 0.262444404272404 | 0.269764975529134 |
| LTR? | 0.235896972771357 | 0.23082925424634 |
| LINE | 0.0765135243686261 | 0.305354675208497 |
| LTR | 0.0644511589032332 | 0.360910107515738 |
| DNA | 0.0636208723784573 | 0.360910107515738 |
| RNA | -0.03001644912745 | 0.969620848217942 |
| scRNA | -0.00861310661197422 | 0.969620848217942 |
| Retroposon | -0.00577735490482679 | 0.969620848217942 |
